# Basic chronobiology: what do sleep physicians need to know?

**DOI:** 10.5935/1984-0063.20200026

**Published:** 2020

**Authors:** Nevin F.W. Zaki, David Warren Spence, Perumal Subramanian, Vijay K Bharti, Ramanujam Karthikeyan, Ahmed Salem BaHammam, Seithikurippu R Pandi-Perumal

**Affiliations:** 1 Mansoura University, Department of Psychiatry - Mansoura - Mansoura - Egypt.; 2 Madurai Kamaraj University, Department of Animal Behaviour & Physiology - Madurai - Tamil Nadu - India.; 3 Independent Researcher,, 652 Dufferin Street, - Toronto - ON - Canada.; 4 Annamalai University, Department of Biochemistry and Biotechnology, Faculty of Science - Chidambaram - Tamil Nadu - India.; 5 Defence Institute of High Altitude Research (DIHAR), Nutrition and Toxicology Laboratory, Defence Research and Development Organization (DRDO), Ministry of Defence - Leh - Jammu and Kashmir - India.; 6 King Saud University, The University Sleep Disorders Center, Department of Medicine, College of Medicine - Riyadh - Riyadh - Saudi Arabia.; 7 Somnogen Canada Inc., Corporate Management - Toronto - Ontario - Canada.

**Keywords:** Chronobiology Discipline, Chronotherapy, Circadian Clocks, Clock Genes, Sleep, Sleep Medicine

## Abstract

Sleep is an essential physiological process, which profoundly affects a wide range of biological activities. It is now known that sleep supports myriad vital functions in the central nervous system. This includes neural plasticity, learning, memory, cognition and emotional regulation. Additionally, it affects basic processes such as cardiovascular, immunological and metabolic activity. Evidence from multiple lines of research has thus shown that good quality of sleep is essential for both survival and optimal functioning of life. Considerable evidence also supports the conclusion that even minimal dysfunctions in circadian regulation can signiﬁcantly disrupt sleep and broadly affect body physiology. As a consequence, it is now appreciated that the therapy of sleep disorders is more complex than was once thought. At present, several clinical disciplines have recognized the signiﬁcance of the biological clock in health and illness, and are incorporating this knowledge into treatment programs. Recent decades have seen the emergence of chronotherapies, i.e., treatment strategies that are aimed at producing adjustments in the circadian clock. The ﬁnal objective of these approaches is to affect basic cellular and physiological processes, which in turn may be at the root of disorders such as physiological aging, immune functioning, metabolic activity, and psychiatric disturbance. It is suggested that the integration of chronobiological perspectives into many mainstream medical disciplines would be of signiﬁcant beneﬁt, both for the reduction of the prevalence of diseases and their treatment. This review considers the physiology of sleep and the importance of timekeeping mechanisms in the regulation of overall health.

## INTRODUCTION

“*Whoever wishes to investigate medicine properly should proceed thus: In the first place to consider the seasons of the year, and what effects each of them produces for they are not all alike, but differ much from themselves in regard to their changes.*”

- Hippocrates, (c. 460 - c. 370 BC)

From the earliest times, there has been a recognition that natural phenomena often manifest in repetitive cycles, and that these are governed by both internal and environmental forces. It was also appreciated that cyclical activity is critical for the functioning of living organisms, including man. In the modern era, this chronobiological perspective is increasingly being reintroduced into the life sciences. It is now known that almost all species possess a genetically programmed timekeeping system, which responds to the environmental rhythms generated by the Earth’s rotation around its axis^1,2^. This endogenous timekeeping system regulates various biological processes including the cell cycle, immunity, metabolism, neural growth and development, reproduction and sleep/wake cycles. Many studies have now shown that adequate sleep is necessary for the proper regulation of vital biological processes as well as for a normal life expectancy^3,4^. Sleep itself however responds to more basic processes. The architectural markers of sleep, including its stages, timing, and duration, are periodic in nature^5^, and these processes, in turn, are driven by the circadian clock, which itself is influenced by a combination of genetic factors, as well as by repetitive events in the environment^6^.

As reviewed below, the various zeitgebers (environmental timekeepers or regulators) of the circadian clock most importantly include ambient lighting, but other events, such as the scheduling of meal times, or occurrences which have important social or psychological significance, can also impact the clock’s circadian phase.

In earlier times, recurrent environmental events were fairly unitary and easy to identify: the rising and setting of the sun, the changing of the seasons, or the migration of animals. In the modern era, however, “timekeeper identification” is more challenging. Humans have become “flooded” with signals reminding them of the passage of time, many of them contradictory and often competitive. The broad array of technological advances that have made our modern-day society possible, e.g., electric “all night” lighting, jet travel, instantaneous news and communication, 24-hour internet access, and many other inventions, have brought with them multiple psychological stresses, as well as a perceived need to accomplish as much as possible in a limited amount of time. This perspective, unfortunately, has also influenced attitudes toward sleep. For many people, this natural biological need is increasingly viewed as a process that takes away from available time that is necessary for “getting things done”. The consequences of this outlook are that, throughout the world, major segments of the population are sleeping less and are living under tremendous “sleep pressure”, i.e., a felt physiological need to make up for prolonged periods of inadequate rest^7^. Over time, sleep deprivation has even broader consequences for human health. It has been suggested that chronic sleep curtailment induces significant stress on pancreatic tissues and that this stress, in turn, promotes the development of serious illnesses such as type 2 diabetes^8^. Further, an increasing amount of evidence suggests that long-term sleep deprivation is a major cause of a range of other clinical abnormalities, including psychiatric^9^, metabolic^10,11^, and immunological disorders^12-14^.

Increasingly, it is being recognized that normalization of disrupted circadian rhythms might be a key approach for treating sleep disorders. This approach in turn depends on a chronopharmacological treatment strategy, which takes into account the entire history of a patient’s disrupted circadian rhythms. This perspective emphasizes the importance of dealing not only with the complications that result from sleep disorders and their immediate biological sequelae, but also with the broader process of managing the environmental and physiological timekeepers of day to day life, factors which can be primary causes of inadequate sleep. The purpose of this review is to summarize what is known about how timing mechanisms influence basic biological processes, and how disruptions to these mechanisms may present in patients who are seen in clinical practice.

### Effect of zeitgebers on the circadian clock

Zeitgebers are the environmental agents, and most often physical, such as the level of ambient light, which serve as timekeepers for the biological clock. These external zeitgebers however can also be linked to events that have social or psychological meaning. The timing of the occurrence of these zeitgebers determines the appropriate regulation of the endogenous oscillator^15^. Therefore, sleep and neurobehavioral processes are deeply affected by the regularity of the entrainment^16^. Light tends to be the dominant zeitgeber for entraining the master clock, the brain’s suprachiasmatic nucleus (SCN), with the daily succession of light and dark periods having been shown to significantly regulate human circadian rhythms^17^. The size and degree of the phase changes of the circadian clock are closely determined by specific time that the zeitgeber occurs. This can then be plotted in a diagram, which, is known as a phase response curve. In clinical applications, manipulation of the time at which light is introduced can be used to either advance or delay the circadian phase. Environmental influences such as degrees of latitude, seasonal changes to the times of sunrise and sunset, continuing exposure to bright artificial light which is kept on until late at night, or if lighting is turned on when the individual is in the early stages of sleep, are all examples of events which could delay the rest phase of the internal clock for 60 min or longer. Conversely, exposure to bright natural or artificial light very early in the morning, before an individual’s normal waking time, can advance the circadian phase for an hour or longer. Finally, light exposure during the middle of the day usually has no effect on the circadian phase^18^.

Recent research has shown that in addition to physical stimuli, more complex, non-photic stimuli can have a substantial entraining effect. The so-called social zeitgebers, such as relationships with individuals, social interaction or working atmosphere can additionally synchronize biological rhythms. Life events, which have a deep psychological impact, such as the death of a family member or loved one, can additionally disrupt the circadian clock. Early studies on the effects of bereavement were in fact focused on the adverse impact that its associated emotions had on the body’s biological rhythms. The initial hypotheses on this point emphasized that rhythm disruptions were the first manifestations of this particular type of life stress^19^. The theory of social zeitgebers was further expanded by Monk and co-workers, who defined social rhythms as habitual behaviors (“social zeitgebers”), which might occur somewhat irregularly (e.g., having breakfast at a certain time on one day and another time on the next day, versus constant timing each day), rather than other behavioral events which have a predictable sequence (for instance, the daily taking of breakfast)^20^.

Taken together, these studies show that the various features of zeitgebers, including their exposure, timing, and duration, can be imbued with psychological significance, which in turn entrain internal circadian cycles, and can thus have a regulatory effect on important biological functions such as sleep.

### Interactive theory of homeostasis and circadian regulation in the process of sleep

The quantity and timing of sleep are regulated by circadian and homeostatic factors, and have been integrated into a theory of sleep known as the two-process model of sleep, which was proposed by Borbély^21^. This model postulates that the need for sleep increases during wakefulness due to homeostatic processes, known as “S”, in the brain, whereas circadian modification is regulated by the circadian process, or “C”.

In a recent reappraisal, Borbély et al.^22^ stated that the conceptualization of process S paralleled recent discoveries regarding topographic differences in brain function. It has been found, for instance, that both increases and decreases in process S are localized in the cortical regions of the brain. Borbély and coworkers have suggested that these regional differences are reflective of changes that occur in slow wave activity, and that such activity can be predicted based on which area of the brain is chosen for analyzing this activity^22^. In the update to their theory, Borbély et al.^22^ have suggested that the two processes are continuously interacting and provided further speculation on the nature of this interaction. This revision of the basic theory is based on the premise that lower circadian amplitude parallels increases in sleep pressure while, conversely, a decline would accompany reductions in sleep pressure. The concept of sleep pressure, which appears to be consistent with evidence regarding the topographical specialization of brain functioning, underscores the importance of the influence of the clock on behavior and physiology.

### Melatonin and sleep

Melatonin is a pleiotropic molecule, which is released by the pineal gland and possesses a broad range of functions^23-25^. Among these functions are its actions in adjusting the timing of the central clock that in turn can produce changes in the sleep/wake cycle^26-28^. Consequently, disruption to melatonin’s activities is likely to produce a broad range of sleep disorders.

Moreover, melatonin can cross the placenta and plays a potent role in synchronizing the fetal biological clock^29^. The synthesis of melatonin is closely affected by ambient light, and can be disrupted if such light is increased above critical levels^30,31^ thus melatonin’s activities represent a complex interplay between physiological need and environmental cues. In addition, to its regulation of the sleep-wake cycle, melatonin closely affects numerous activities throughout the body. These include, but are not limited to, blood pressure and autonomic regulation, and immune system regulation, which is accomplished through enhanced production of cytokines and interleukins. Additionally, melatonin has a role in obesity and control of energy expenditure. Other melatonin effects include detoxification of free radicals and antioxidant actions, which can protect the gastrointestinal tract from ulcers^32^. These broad-ranging effects underscore melatonin’s significance as a major regulator of bodily activities.

### Human circadian rhythm sleep disorders (CRSDs)

The International Classification of Sleep Disorders (ICSD-3) redefined this group of sleep disorders into “sleep-wake” disorders to underscore the importance of the physiologic impact, which occur throughout the 24-h cycle.

Circadian rhythm sleep-wake disorders occur when the internal circadian clock becomes desynchronous due to endogenous dysfunctions, or when it is disrupted by exogenous factors in the environment. These include delayed sleep phase disorder (DSPS), advanced sleep phase disorder (ASPS), irregular sleep-wake rhythm disorder, non-24-h sleep-wake rhythm disorder, jet lag disorder, and shift work disorder^33^. Among the symptoms of clock desynchronization are atypical clinical symptoms, including persistent fatigue, chronic insomnia, poor appetite, or mood disorders. Nevertheless, some cases of desynchronization do not manifest in any of these clinical signs^26^.

### Delayed sleep phase disorder

One of the most common of the circadian rhythm sleep-wake disorders (CRSDs) is delayed sleep phase disorder (DSPS), a condition that is often mistaken for sleep initiation insomnia. The DSPS typically is a common complaint that emerges during teenage years, but may also persist into adulthood. Affected individuals find it difficult to initiate sleep at an appropriately early time, and, additionally, may have difficulty in rising at a desirable time in the morning. The consequence of these problems is a chronic and often quite severe sleep restriction; all resulting from efforts adhere to socially expected waking schedules. The resulting sleep debt tends to drive compensatory efforts to obtain extra sleep on weekends and free days. Despite the obvious inconvenience, which the disorder poses to affected individuals, the associated sleep quality and duration are essentially normal but are simply delayed^34^.

### Advanced sleep phase disorder

ASPS is characterized by major advances in the major sleep period in which the individual habitually and involuntarily initiates sleep and experiences wakeup times that are considerably earlier than the desired clock time. This condition is found more frequently in middle aged and older adults. Affected individuals complain of sleepiness in the late afternoon or early evening and find it difficult to stay asleep during the early morning hours. Most patients with this condition report that they have a sleep onset occurring between 6pm and 9pm, and tend to awaken between 2am and 5am. If the sleep time is restricted because of social or occupational obligations, individuals with ASPS continue to wake up at a time that is earlier than desired and consequently resulting in sleep deficiency over a prolonged period^35^.

### Irregular sleep-wake rhythm disorder

Patients who experience multiple periods of sleep within a 24-hour period are referred to as having an irregular sleep-wake rhythm disorder. Affected individuals show symptoms of insomnia, which may include difficulties with sleep initiation or sleep maintenance, and excessive sleepiness during the day. The condition affects many categories of patients, including children with neurodevelopmental disorders but occurs particularly in older adults with neurodegenerative disorders^36^.

Sleep-wake rhythm disorders (SWRD’s) can also occur at irregular times, i.e., in periods other than a 24-h day. Any condition in this category is deemed to meet these criteria according to ICSD-3:

1. A history of insomnia, excessive daytime sleepiness, or both, which alternate with asymptomatic episodes, due to misalignment between the 24-h LD cycle and the non-entrained endogenous circadian rhythm of sleep-wake propensity;
2. Symptoms, which persist over the course of at least 3 months;
3. A complete diagnosis is based on data from sleep diaries and actigraphy measurements for a minimum period of two weeks. In the case of blind individuals a longer duration of testing is required and actigraphy records must show that the sleep/wake episodes occur daily. Further, the circadian period is longer than 24-h and is not better explained by another current sleep disorder, medical or neurological disorder, mental disorder, medication use, nor by the presence of a substance use disorder^37^.

### Jet lag disorder

Another condition is jet lag disorder (JLD), which is caused by circadian misalignment due to crossing time zones too quickly for the adjustment of circadian system. This may result in difficulties for circadian system resynchronization, depending on how many time zones were crossed, and the direction of travel. Readjustment of the circadian system may require several days in severe cases. Other factors which can exacerbate the severity of jet lag symptoms include characteristics of the local environment, including the saliency and availability of local time cues. Intra-individual factors such as the ability to sleep while sitting upright in an airline seat or individual differences in adaptability to phase changes can also contribute.

### Shift work disorder

An increasingly common phenomenon in industrialized societies around the world is the prevalence of work assignments occurring in the nighttime hours. Such work scheduling is now known to produce a condition known as shift work disorder (SWD). Night work assignments can result in excessive sleepiness during the day, or inability to sleep when sleep time is allowed. To meet the criteria for this condition, the symptoms must not be the result of any other sleep disorder, medical condition, nor of another medication effect. More than 20% of shift workers have symptoms of SWD. The most salient consequence of this disorder is inattentiveness and a decline in cognitive efficiency. These symptoms in turn may lead to an increase in slip and fall injuries, mistakes, or increases in industrial accidents. Furthermore, additional accidents may occur to shift workers who, at the conclusion of their shift, are driving home from work in the early morning hours, a period which coincides with their lowest level of alertness. This combination of risk factors has been found to increase the likelihood that affected individuals will have a motor vehicle collision^38^. Other long-term effects that have been found to be associated with shift work are an increased susceptibility to various cancers^39^, sleep disturbances^40^, gastrointestinal problems^41^, neuropsychological issues and cardiovascular symptoms^42^.

These symptoms may be linked to desynchronization between the internal clock and the LD cycle. It has been recommended that shift workers should avoid exposure to light for 30 min prior to going to sleep following their work assignments. They should also avoid taking an additional morning shift assignment that starts before 7am or to work for more than three successive night shifts. Additionally, women who are pregnant should avoid performing night shift work^43^. It has been found that glasses that restrict light in wavelengths of less than 680 nm, i.e., in the “blue” end of the visual spectrum, may reduce the severity of these symptoms^44^.

### Clock genes and sleep

Many research reports have established that clock genes are expressed in several brain regions, which are involved in sleep regulation, i.e., of its stages, structure and duration^45^. Several animal model studies have shown that the homeostatic component of sleep is substantially controlled by the circadian clock. The absence of CLOCK in mice was found to significantly alter the sleep organization and particularly, it reduced NREM sleep around 60 to 120 min compared to the control mice^46^. Deletion of BMAL1 results in a change in sleep architecture and sleep duration in mice, and the BMAL1 mutant mice exhibited an increased duration of NREM sleep^47,48^.

In mice, genetic mutation of both Cry1 and Cry2 resulted in abnormal sleep stages and structures producing an enhanced duration of NREM sleep than in the wild type mice ^49^. These findings thus suggest that clock genes have a functional significance in the regulation of sleep homeostasis processes.

Dysregulation in the expression of circadian clock genes in the hypothalamic suprachiasmatic nucleus (SCN) has been shown to be associated with disrupted circadian activity. Per3 polymorphism studies have shown positive correlations with preferences for morningness and eveningness^50,51^. Genetic variation recognized at Clock, Per2, Per3, and Npas2 has been found to be associated with the sleep/wake scheduling of seasonal affective disorder (SAD) patients^52^. The condition known as familial advanced sleep phase syndrome (FASPS) has been found to be associated with a polymorphism identified at the locus of Per2^53^ and CKIδ^54^. The creation of a mutation at the locus of CKIδ in mice produced a reduction in the circadian period^54^. In drosophila, the experimental generation of the same mutation produced a lengthening of the circadian period^92^. Alleles identified at Per3 gene was found to influence the prevalence of DSPS^55,56^. In bipolar patients, clock mutant was correlated with delays in their sleep phase for 79 min and the patients showed a reduction in their sleep length of 1.25 h^57^. Taken together, this evidence demonstrates that clock genes are either directly or closely involved in the timing, duration, architecture, stages and homeostatic mechanisms of sleep.

### Chronotype and sleep

Based on the preference of behavioral attributes of sleep/wake schedule, individuals are classified into different phenotypes referred to as morning types (larks) and evening types (owls). Morning type individuals prefer to sleep early in the night and to awaken early in the morning, whereas evening type individuals prefer to go to sleep late in the night and to awaken late in the morning. In 1976, a questionnaire was developed to assess these preferences^58^. Individuals could thus be categorized into morning, evening and intermediate types depending on their activities and scoring pattern of the questionnaire^58^. Due to variations in genetic machinery, a wide distribution exists in the occurrence of chronotypes. Questionnaire testing has revealed the existence of individuals (“subtypes”) with preferences for the moderate morning, extreme morning, moderate evening, extreme evening and intermediate waking times^15,58^. The chronotype is influenced by genetic vulnerability^50,53,59^, environment^60^, age and gender^15,61^. Morning type and evening type individuals have been shown to exhibit significant differences in their rectal temperatures and subjective alertness^59^. In addition, the sleep time of morning chronotypes has been shown to undergo advances of at least 80 min^62^.

Most children are categorized as early chronotypes, an indication that their circadian clock is advanced. The main characteristic feature of adolescence is the ability to stay awake until late at night and then to start to sleep in a bed^15^. There is a progressive change in the physiological functioning of the circadian clock, and by age of 20, sleep timing tends to advance from the late status^15^. In a population study comprising 2135 university students, gender- based differences between men and women were identified. The authors found that men have a greater preference for evening times whereas women preferred morning time^61^. Some limited evidence has suggested that the sleep/wake cycle of short sleepers and evening types are affected when social time schedules are changed to daylight saving time^63^. In an analysis of 21, 600 chronotypes, it has been found that solar cues (sunlight intensity and duration) are more important in determining the entrainment of the human circadian clock than the social timing schedule of the society^60^. It has been suggested that the preference for morningness and eveningness is caused by the phase of the circadian clock rather than by individual behavioral activity^64^. It should be noted that both schizophrenia and bipolar patients have a scoring pattern more associated with eveningness preferences than control subjects^65^. These findings suggest that efforts, for instance, to improve efficiency in the work environment or to treat clinically aberrant conditions, require an awareness of the phase of affected individuals’ circadian clocks.

### Modern lifestyles and circadian clock complications

Modern technological devices such as portable telephones, computers with light-emitting screens, and numerous other devices can negatively impact sleep quality. This is particularly the case when they are used late at night inasmuch as the associated light exposure can affect the phase and amplitude of the melatonin cycle, thus resulting in poor quality of sleep and reduced sleep duration. Numerous studies now show that simulated monochromatic light at late nighttime hours affects the synthesis of melatonin, a major feedback regulator of circadian clock^66^. The associated suppression in melatonin levels produces increased alertness, which in turn can impair sleep length and quality^67-69^. A study has demonstrated that reading with the help of light-emitting electronic (LEE) devices delays the secretion of melatonin for 1.5 h^69^. In addition, people who have used LEE devices for reading reported delays in their sleep onset and poor sleep quality in the evening when compared to those subjects who read with the printed version on paper^69^. The prevalence of jet lag in individuals has provided researchers with an opportunity to study the internal clock in humans. Evidence from numerous studies in this area strongly points to the need for appropriate entrainment with the environmental cues^70^.

Misalignment of the circadian system can adversely affect sleep/wake cycles, which in turn can have broader effects on overall health. Behavioral choices affecting sleep times are major contributors to this process. Prolonged shift work can accelerate the development of type 2 diabetes (T2DM)^71,72^. In addition, individuals having a long-term circadian disruption are vulnerable to psychiatric disorders^16,73,74^, metabolic disorders, including obesity^75,76^, and T2DM^77-83^, cardiovascular disorders^84,85^ and cancer^86,87^. In individuals who work rotating shifts, adaptation to the new work schedule is complicated by the fact that they encounter natural light exposure while they return to the home in broad daylight. This, in turn, can interfere with the entrainment of the circadian clock, and often leads to circadian disruption. Those who are assigned to work rotating shifts often have problems in adapting to a new and unaccustomed sleep schedule. Modern society has provided many benefits that have reduced the difficulties of living that prevailed for many centuries previously. These benefits have come at a cost, however, inasmuch as trends such as shift work, jet travel, the availability of nighttime shopping and entertainment, and other pressures of our 24/7 society, are now known to interfere with the natural functioning of the circadian system. These consequences have unfortunately increased the risk of health problems for individuals as well as adding to the costs of public health care.

### Mealtime and circadian rhythm

Cumulative evidence reveals that mealtime closely interacts with the circadian clock^88^. “Chrononutrition”, which means meal timings, is a recently proposed specialty that focuses the interaction between mealtime, circadian clock and metabolism. Additionally, mealtimes affect metabolism and body weight^89^. Eating at the wrong time of day results in a misalignment between the peripheral circadian clocks and the central biological clock in the SCN. The resultant desynchronization enhances the risk of developing cardiometabolic disorders^88,90^. Nocturnal species consume most of their daily food requirement at night. For example, mice eat most of their daily consumption of food (70-80%) during the dark half of the day (active phase)^91^. Therefore, when mealtime is limited to the light phase (inactive phase) of the day, uncoupling between peripheral and central clocks takes place, and , in as little as one week, mice put on more weight compared to their counterparts fed during the dark phase in as little as one week^92^. Moreover, one more study in mice revealed that confining food accessibility to the active phase (8-9 h) was protective against weight gain and metabolic syndrome, secondary to atherogenic food ingestion ^93^. The benefical effect of confining food to the active phase is not due to to caloric restriction. Hatori et al.^94^ subjected mice to either an ad libitum dietary access or constrained their feeding time to 8-h per day (active phase) of a high-fat-diet. Intriguingly, mice with limited feeding time ate similar calories to their counterparts with ad libitum food access, yet limiting food access to 8 h had a protective effect against weight gain, increased insulin levels, hepatic steatosis, and systemic inflammation^94^.

Likewise, in humans, previous reports have demonstrated comparable outcomes, where eating at the wrong time (nighttime “in active phase”) was accompanied with a greater risk of developing cardiometabolic dysfunction^90^. In a Swedish study of 3610 participants of both sexes, eating late at night was associated with an increased obesity odds ratio (OR) of 1.62 (95% confidence interval [CI], 1.10-2.39) compared to those who did not eat late at night^95^. A recent systematic review and meta-analysis of 10 observational and experimental studies that evaluated the impact of mealtime on obesity and metabolic changes in humans demonstrated a negative influence of late mealtime on weight and metabolism^96^. Furthermore, both observational and experimental studies demonstrated a link between late meal timing with hyperglycemia and diabetes mellitus^96^.

### Chronotherapy

Chronotherapy refers to the use of treatments that are sensitive to the circadian characteristics of the patient. The larger purpose of this effort is to enhance patients’ therapeutic responses, e.g., treatment of sleep and psychiatric disorders with either light therapy or melatonin^97^.

Chronotherapy focuses on the degree of synchronization of a patient’s rhythmic cycles. These include circadian parameters such as sleep/wake cycles, as well as amplitude changes in cortisol, melatonin and body temperature^26^. Among the tools of chronotherapy are, e.g., the time of sampling of bodily fluids, the timing of therapy, and regular assessment of therapeutic responses. The goal of chronotherapy is to restore the functional organization of clock genes by the proper scheduling of zeitgebers and the avoidance of factors that may disrupt the clock. A major focus of chronotherapy is thus to manage the effects of zeitgebers on biological functioning, all with the purpose of strengthening the weakened circadian clock^98^.

It is known that bright white light can entrain and shift human circadian rhythms. When used for this purpose it is called a ‘chronobiotic’ (i.e., an agent or a substance that corrects the timing or strengthens oscillations of the master biological clock)^99,100^.

Treatment involving bright light exposure is now used clinically and experimentally for treating sleep disorders and to help older patients whose circadian rhythms have been disrupted^101^.

The application of strategically timed exposure to bright light has been used to assist shift workers in adjusting to night shift work inasmuch as it has been found to be effective for alleviating sleepiness and fatigue.

Disorders such as sleep phase advances or delays, which directly reflect disruptions of the biological clock, can be remediated by administration of light treatment and sleep restriction. These treatments can also be applied to the enhancement of mood states in patients with psychiatric disorders^102-104^. In the treatment of DSPS, the sleep phase can be advanced by strategically exposing patients to bright light at specific times in the morning over a period of two weeks (2500 lux, 2 h duration within a 3 h period between 6:00 and 9:00am). During the same treatment program the use of specially developed goggles (from 16:00 to dusk and only bedside lamps from dusk to bedtime), which restrict exposure to light below the 680 nm range, can attenuate the melatonin-suppressant effects of nighttime light exposure.

The recovery process from jet lag can similarly be accelerated up by a planned regimen of timed exposure to sunlight^105^. Sleep hygiene and management of the LD and sleep schedule are the behavioral techniques that can be adopted. Re-entrainment of desynchronized rhythms uses both behavioral and non-behavioral strategies. Insomnia and excessive daytime sleepiness can be treated with several pharmacological agents while melatonin is used to realign circadian rhythms and thus to promote normal sleep^28^. Exposure to bright light in the evening can rapidly delay the circadian system, and thus has been used for the treatment of patients suffering from ASPS.

A growing amount of evidence has confirmed that disruptions to the circadian system can contribute to mood disorders. Increasingly patients with depressed mood and related disorders are being treated through programs aimed at realigning the internal clock^19,106,107^. Regulation of sleep/wake schedule has been shown to improve the mood profile of bipolar disorder patients^108^. In a number of psychiatric and neurodegenerative diseases there are often disruptions to the sleep-wake cycles, and it has been reported that light therapy has been used to address these problems^109,110^. Light is considered to be one of the best antidepressants due to its healing effect on mood deterioration and thus it is considered as a treatment of choice for ameliorating seasonal affective disorder (SAD)^107,111^.

### Chronopharmacology

Chronopharmacology deals with how drugs influence the circadian rhythms of individuals, and has the further purpose of identifying the specific times at which a drug has its optimal level of efficacy. The effects of the scheduled administration of psychoactive drugs at various times have been examined in more detail^112^. Dawson and Armstrong, in 1996^99^, following a review of the extant evidence, concluded that how a sleep disorder manifests is connected to the phase-shifts of the circadian system. This view thus pointed to the importance of the scheduling of drug administration. Clomipramine has been found to provide the best response when administrated around mid-day, while the side effects are greatest when it is administrated before bedtime^113^.

Whether or not doses of neuroleptics are lethal has been shown to be reliant on the time of administration^114^. The effectiveness of clinical therapies has been found to be critically dependent on identifying the location of action of chronobiotics within the circadian system.

The circadian target sites chosen are in turn dependent on the specific physiological objective of the therapy. These sites may be at the level of several functional areas including: (a) input systems to the master oscillator, (b) the master clock itself, (c) the entrainment mechanism, (d) coupling pathways, (e) slave oscillators, (f) passive systems, or (g) feedback via overt rhythms in the retina by increasing or decreasing the sensitivity to light. In the retinohypothalamic tract (RHT) these manipulations can modulate the neurotransmitter glutamate and acetylcholine, which in turn can phase shift the circadian timing. The geniculohypothalamic tract (GHT) is modulated by neuropeptide Y while the SCN is stimulated by drugs acting on GABA and arginine vasopressin^115^.

These findings demonstrate that treatment effectiveness for a range of disorders can be enhanced when therapy selection for sleep disorders is based on the identification of patients’ chronotypes. It is now evident that, before treatment is attempted, the patients’ circadian functionality should be closely assessed and monitored. The goal of such a diagnostic approach would include the tracking of the activity of melatonin and its associated biochemical effects.

Taken together, these findings support the conclusion that the treatment of sleep disorders can be improved and made most effective when the dynamic state of patients’ circadian timing systems are assessed and are made the focal determinant of how therapy programs are developed.

### Melatonin as a chronobiotic drug

Due to its influence on metabolic and electrical activity in the SCN, melatonin is considered to be a chronobiotic drug^32^. Other studies have shown that repeated administration of exogenous melatonin can entrain the rest/activity cycle, possibly through its effect as a “dark pulse”^99^. Lewy et al.^116^ found that after four consecutive days of oral administration of melatonin the DLMO (the first surge of melatonin output in the evening) was altered.

When administered in the evening and the early half of the night, melatonin has been found to phase advance the endogenous clock, whereas, if given during the second half of the night, the phase of the endogenous clock is delayed. It has been hypothesized that adjustments to the circadian phase depend on the timing of melatonin administration, which is either advanced or delayed^116^. Among the elderly, or in those who are chronically exposed to environmental stressors, endogenous melatonin levels are typically at sub-optimal levels, a physiological state, which is often associated with insomnia. In clinical cases such as these, exogenously administered melatonin can be of benefit. Melatonin is also known to ameliorate the deleterious effects of rotating shift work, including poor sleep quality and circadian disruption.

Other associated studies have argued that melatonin hinders the elevation of core body temperature, which is responsible for reducing sleep duration^117,118^. Melatonin, which is now available in most countries as a non-prescription nutrient, thus offers a convenient means for regulating the circadian clock, thereby providing shift workers, particularly those who must work rapidly rotating shifts, the option to enhance their sleep quality and duration. Among those who work slowly rotating shifts, and thus who may experience more severe circadian disruption, it is recommended that bright light exposure be combined with administration, of melatonin which has been found to have additional protectant effects.

## CONCLUSION

The evidence reviewed above supports the conclusion that the circadian clock plays a prominent role in the onset, structure, timing and duration of sleep. These findings indicate further that the misalignment of the internal clock can produce significant sleep abnormalities.

Further, it is evident from the available evidence that identifying sleep abnormalities may represent an important first step in the pathway of diagnosing other physiological or psychiatric disorders. It is also obvious that sleep disorders may not only trigger the development of other more complex illnesses but also represent an accessible means for preventing these disorders. Many sleep disorders are preventable and often result from lifestyle choices. In an era of artificial lighting systems, irregular sleep/wake schedules, and “high pressure” living, all of which may be sustained for weeks or even years, there is an urgent need to prioritize chronobiological perspectives in medical care delivery as well as in public education. The effect of artificial light from light emitting electrical (LEE) devices, especially when these are used late at night, is widely regarded to be innocuous. In fact such devices can significantly disrupt the circadian clock and interfere with the total process of sleep. Many of the latest technological instruments warrant critical guidelines for general use, and public health warnings should be issued regarding their risks to human health.

Guidelines could be prescribed for environmental lighting in workplaces, or for the scheduling of shift work. These measures may increase the ability of shift workers to adapt to work requirements. This will be of benefit not only for the health status of workers but could help to cut costs to employers by reducing sleepiness-related accidents, impulsivity, or poor decision making at the job site. One study has demonstrated that workers on rotating shiftsworkers were able to adapt to new schedules with fewer difficulties when they avoided natural LD cycles^119^. Long-term studies are required to study the impact of circadian clock disruption and its influence over sleep/wake processes^120,121^. Moreover, more detailed and extensive studies should be carried out to describe the effect of artificial lighting on the circadian clock and how it affects the timing, architecture, quality and duration of sleep. Chronotherapy is gaining some attention but is still at a preliminary stage of development. The present authors conclude that greater interest and effort should be taken to incorporate this therapy into more widespread use and that success in this endeavor will be broadly beneficial. The adoption of chronotherapeutic perspectives would represent an important step not only for medical therapy but also for the prevention of illness, and thus would thus represent a major advance in healthcare delivery.

## Figures and Tables

**Figure 1:**
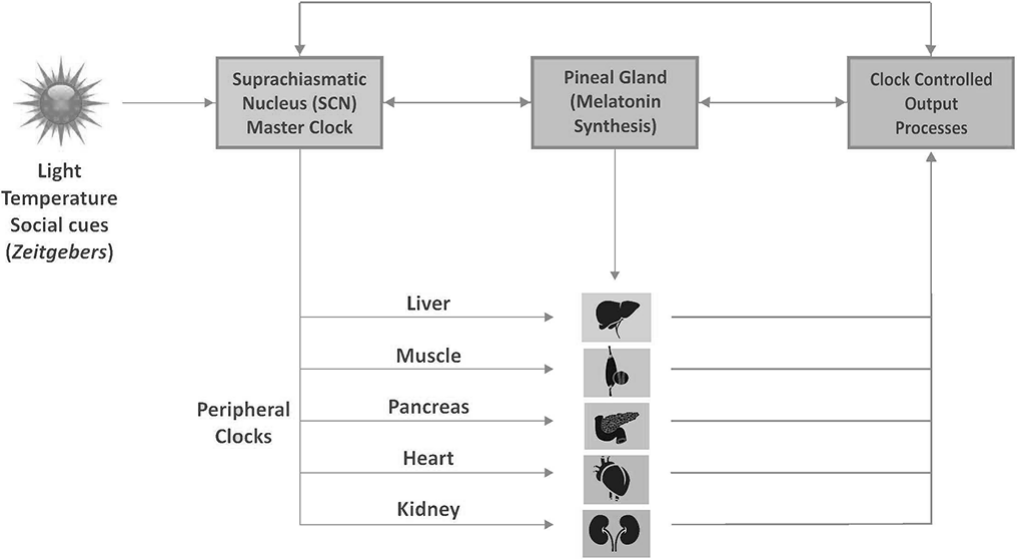
The schematic diagram shows the components and the outline of organization of circadian timing system in humans. Zeitgebers (light, temperature, and social cues) provide the environmental information to the suprachiasmatic nucleus (SCN; ‘master oscillator’) through melanopsin-expressing retinal ganglion cells (RGCs), which are intrinsically photosensitive (ipRGCs). From the SCN, the information passes to the peripheral clocks through humoral and neural molecules located in various organs including liver, skeletal muscle, pancreas, heart, and kidney. By this way, numerous biochemical, molecular, cellular and behavioral processes are scheduled and coordinated by the circadian clock system.

**Figure 2:**
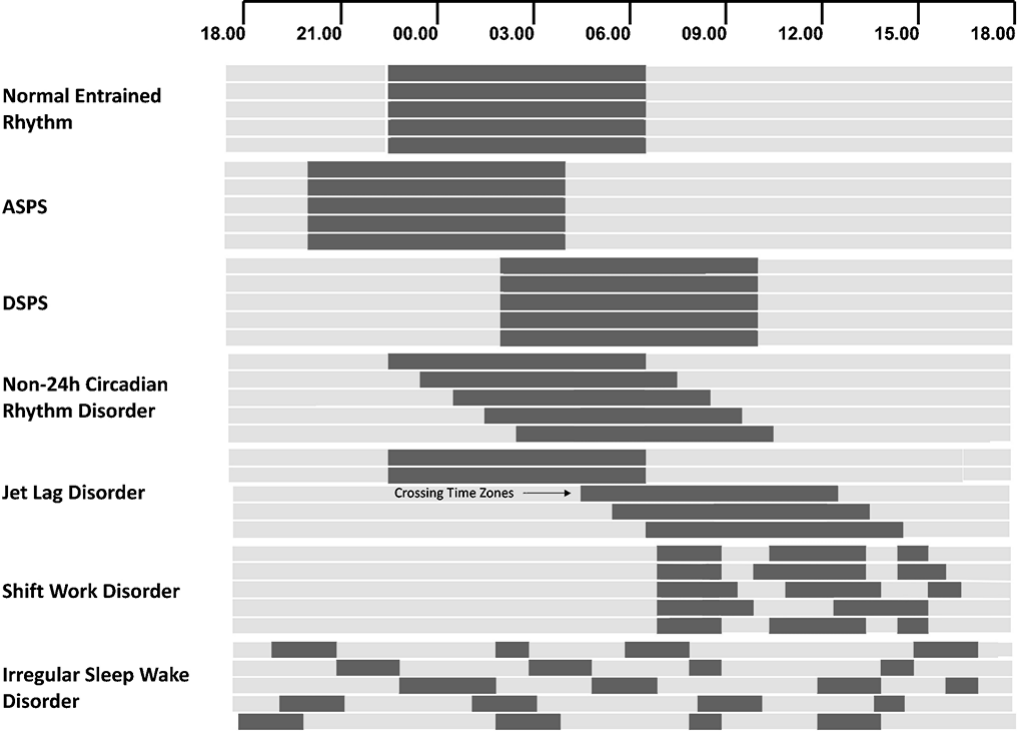
This pictorial representation shows the types of circadian rhythm sleep disorders (CRSDs) in humans which arise due to the phase alteration of the circadian clock. From the top, the first representation shows normal entrained sleep/wake cycle (~24h) synchronized to the environment (LD cycle). In ASPS patients, the sleep phase is advanced than the conventional sleep time and individuals go to bed early and wake early in the early morning. In DSPS patients, the phase of the sleep is rather delayed and in both these disorders, patients have difficulties in synchronizing their activities to the social schedule of the society. In non-24 h circadian rhythm disorder, the patient’s rest/activity cycle is delayed every day and this results in sleep onset problems and persisting sleepiness during daytime. In subjects with shift work disorder, individuals work during the night, which is the circadian time designated, for sleep and consumption of food, and exposed to light at an inappropriate time (e.g. LAN; Light At Night). This abnormal behavioral disorder leads to impairment in cognition, attention, and sleep. Moreover, they are prone to work-related injuries, road, and occupational accidents. When individuals travel to a different time zone, circadian clock takes some time to adjust to the new time schedule. Until the adjustment of the clock, individuals have difficulties in getting sleep and other behavioral activities leading to jet lag. In irregular sleep-wake disorder, the patients exhibit a characteristic lack of unconsolidated sleep/wake pattern and possess at least three bouts of sleep.
